# Protocol for the Arterial Revascularisation Trial (ART). A randomised trial to compare survival following bilateral versus single internal mammary grafting in coronary revascularisation [ISRCTN46552265]

**DOI:** 10.1186/1745-6215-7-7

**Published:** 2006-03-30

**Authors:** David P Taggart, Belinda Lees, Alastair Gray, Douglas G Altman, Marcus Flather, Keith Channon, the ART Investigators

**Affiliations:** 1 Nuffield Dept of Surgery, University of Oxford, John Radcliffe Hospital, Oxford, OX3 9DU, UK; 2 Clinical Trials and Evaluation Unit, Royal Brompton and Harefield NHS Trust, Sydney Street, London SW3 6NP, UK; 3 Health Economics Research Centre, Institute of Health Sciences, Oxford OX3 7LF, UK; 4 Cancer Research UK Medical Statistics Group, Centre for Statistics in Medicine, Wolfson College, University of Oxford, Linton Road, Oxford OX2 6UD, UK; 5 Department of Cardiovascular Medicine, John Radcliffe Hospital, Headington, Oxford OX3 9DU, UK

## Abstract

**Background:**

Standard coronary artery bypass graft surgery uses a single internal mammary artery and supplemental vein or radial artery grafts. Several observational studies have suggested a survival benefit with two internal mammary artery grafts compared to a single internal mammary artery graft, but this has not been tested in a randomised trial. The Arterial Revascularisation Trial is a Medical Research Council and British Heart Foundation funded, multi-centre international trial comparing single internal mammary artery grafting versus bilateral internal mammary artery grafting.

**Methods/Design:**

Twenty centres in the UK, Australia, Poland and Brazil are planning to randomise 3000 coronary artery bypass graft surgery patients to single or bilateral internal mammary artery grafting. Supplemental grafts may be either saphenous vein or radial artery. Coronary artery bypass grafting can be performed as an on-pump or off-pump procedure. The primary outcome is survival at 10 years and secondary end-points include clinical events, quality of life and cost effectiveness. The effect of age, left ventricular function, diabetes, number of grafts, vein grafts and off-pump surgery are pre-specified subgroups.

**Discussion:**

The Arterial Revascularisation Trial is one of the first randomised trials to evaluate the effects on survival and other clinical outcomes of single internal mammary artery grafting versus bilateral internal mammary artery grafting, and will help to establish the best approach for patients requiring coronary artery bypass graft surgery.

## Background

Coronary artery bypass graft (CABG) surgery is the optimal therapy, prognostically and symptomatically, for multi-vessel ischaemic heart disease [1]. Worldwide, around 800,000 CABG are performed annually. Recognising the under provision of CABG in the UK, the National Service Framework (NSF) aims to increase numbers from 500 [2] to 750 per million of population. The requirement for CABG is also likely to increase because of a growing elderly population with coronary disease and because percutaneous interventions ultimately lead to an increased requirement for CABG [3].

**Figure 1:**
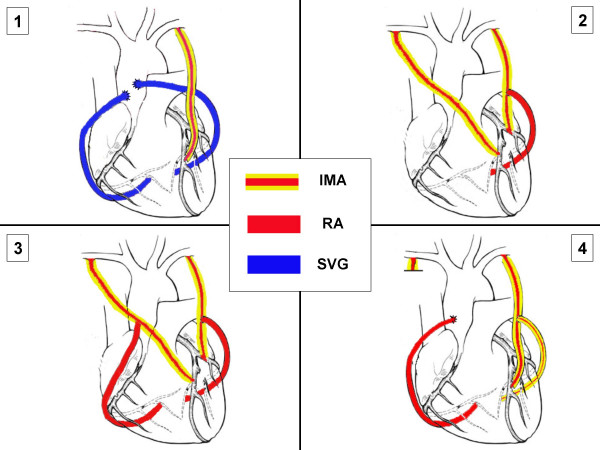
Schematic drawing showing typical examples of: (1) Single internal mammary artery (SIMA) grafts and (2–4) Bilateral internal mammary artery (BIMA) grafts. Key: IMA = internal mammary artery; RA = radial artery; SVG = saphenous vein graft.

Most CABG patients require three grafts and the standard operation uses a single internal mammary artery (SIMA) and supplemental vein or radial artery grafts (Figure 1). CABG provides excellent short and intermediate term outcomes but its long-term efficacy is limited by vein graft failure. Ten years after CABG around 1/2 of vein grafts are blocked and of the remaining 50% half are severely diseased [6] whereas up to 95% of internal mammary artery (IMA) conduits remain disease free. In addition to reducing long-term survival, vein graft failure significantly increases the risk of recurrent angina, late myocardial infarction and the need for further intervention [4-7]. Indeed, by 10 years after CABG >50% of patients have recurrent angina and up to 1/3 may eventually require redo CABG [8-11]. Aspirin and statins [10,11] may improve vein graft patency but are unlikely to achieve the patency rates of IMA grafts.

As discussed below, bilateral IMA (BIMA) in comparison to SIMA grafts, may improve survival and reduce the need for redo CABG. However while BIMA grafting is common in some centres in Europe, America, Australia and Japan, it still not widely used. For example, in 1998 15% of UK CABG patients received two arterial grafts (and a significant proportion of these would have used a radial artery rather than a second IMA graft) [2]. The major reasons for not using BIMA grafts is because of no definitive evidence of benefits (there are no randomised trials) and the perceptions that it is technically more challenging, more prolonged and increases the risk of impaired wound healing. Given the number of CABG procedures currently performed in the UK and the aim of the NSF to increase these numbers, it is also important to obtain accurate information on the costs and cost-effectiveness of using BIMA versus SIMA grafts.

Nine studies, reviewed extensively in reference 12, have compared the influence of SIMA and BIMA grafts on survival and the need for redo surgery. Interpretation of individual studies is, however, complicated by lack of randomisation, small patient numbers and inadequate length or completeness of follow-up. Furthermore, as the use of BIMA grafts was initially confined to younger, lower risk patients, any long-term benefits were attributed to the inherently more favourable characteristics of these patients, obscuring any benefit of BIMA.

**Figure 2:**
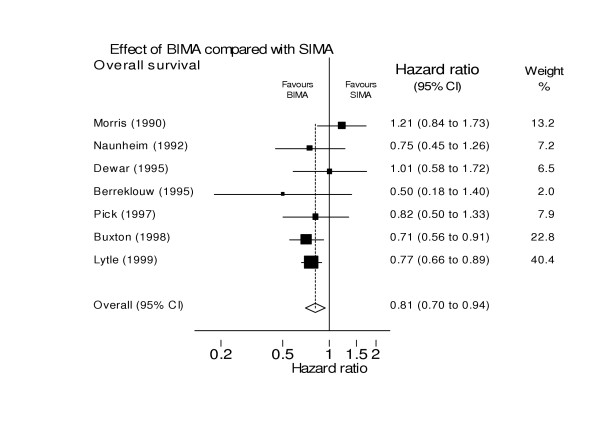
Survival following bilateral internal mammary artery (IMA) grafting compared to single IMA grafting [12].

A recent systematic review was performed of those studies, meeting pre-specified criteria relating to patient selection, comparability of groups, outcome assessment, and completeness of follow-up, to determine if there are differences in survival, expressed as a pooled hazard ratio (HR), in patients receiving SIMA or BIMA [12] (Figure 2). The analysis included 15962 patients comprising 11269 SIMA and 4693 BIMA patients from seven studies that either matched or adjusted for age, sex, ventricular function and diabetes. The results suggested a survival advantage for BIMA grafts (HR for death = 0.81, 95%CI: 0.70 to 0.94) without any evidence of a detrimental effect, however there was very limited evidence relating to secondary endpoints including possible adverse consequences.

In the largest single study [13], of 8000 SIMA and 2000 BIMA patients, Lytle *et al *reported that the HR for death was decreased by 23% in the BIMA group at 12 years and the need for redo CABG reduced from 40% in the SIMA to 10% in the BIMA group.

In another observational study, published after the systematic review, Endo *et al *reported outcome in 688 SIMA and 443 BIMA patients [14]. The groups were similar regarding age and ventricular function but there were more diabetics in the BIMA group (18% vs 13%) and females in the SIMA group (19% vs 10%). At six years the combined incidence of death, myocardial infarction and redo CABG was lower in the BIMA group (p = 0.06) and particularly in the 782 patients below 71 years and with an ejection fraction > 0.4 (HR: 0.61; 95%CI, 0.38 to 0.98:p = 0.04). As vein graft failure increases markedly beyond seven years after CABG the authors suggest that the benefits of BIMA grafts are likely to increase with further follow-up.

Two studies reported no benefit of BIMA grafting [15,16]. Sergeant's study, however, had fewer than 100 BIMA patients with 10-year follow up and use of the second IMA was frequently suboptimal [15]. Kurlansky et al reported no survival difference at ten years in 261 women with BIMA grafts and a matched cohort with SIMA grafts [16]. However, 81% of the BIMA group vs 66% of the SIMA group had triple vessel disease (p < 0.001) and only 120 patients in each group were available for comparison at ten years.

For optimal patency both IMA should be placed to the left sided arteries [17-19] (Figure 1). Patency of the right IMA is reduced if used as a free aortic graft [18] or placed to the right coronary artery [19] due to size discrepancy and eventual disease development at the crux.

Angiographic studies demonstrate markedly superior patency of BIMA grafts, compared to vein grafts, refuting the assertion that the superior patency of IMA grafts is due to better 'run-off' in the LAD territory. Patency rates for BIMA to various coronary arteries, are 98% at 7 days [14] and 95% at two [20] and seven years [21]. Furthermore, off-pump CABG (OPCAB) now makes CABG feasible in patients whose advanced age previously precluded CABG using cardiopulmonary bypass. A composite radial artery from one or both IMA, allows up to four grafts as an 'off-pump' CABG, eliminating both cardiopulmonary bypass and aortic manipulation and minimizing the risk of cerebral injury [22].

Opposition to BIMA grafting is largely based on the perception of increased perioperative risk and especially sternal wound morbidity. There is consistent evidence, however, that the operative mortality of BIMA grafting at 1%–2% [12,13] is no higher, and may, in fact, be less than that of the standard operation because of a reduced risk of early graft failure.

Sternal dehiscence is a potential complication of BIMA grafts and particularly in diabetics. In reality, there is only a minimal increase in the risk of impaired wound healing in both non-diabetics [13,23-27] and diabetics [23-27] unless the patient is morbidly obese and/or has marked respiratory impairment [24]. The risk of impaired wound healing can be minimized with judicious patient selection and modification of the IMA harvesting method whereby a 'skeletonized' rather than 'pedicled' fashion preserves collaterals and sternal blood supply [25] and improves wound healing, particularly in diabetics. No difference has been found in myocardial enzymes [28], parameters of respiratory exchange [29] or in respiratory injury between SIMA and BIMA patients. BIMA harvesting adds around 30 minutes to a three-hour operation.

Uncertainty remains because there is no randomised evidence, therefore a randomised trial – the Arterial Revascularisation Trial (ART) – has been designed to compare SIMA versus BIMA grafting in coronary revascularisation. ART will compare survival rates, need for redo CABG, other clinical events, quality of life and cost effectiveness of SIMA versus BIMA grafting.

The main objective of ART is to assess whether the use of both IMA during CABG (BIMA) improves survival and reduces the need for further interventions over that observed with a single IMA (SIMA).

## Methods/Design

### Trial design

Two-arm, randomised multi-centre trial. Patients will be randomised to SIMA or BIMA with supplemental vein or radial artery grafts as required.

### Eligibility

#### Inclusion criteria

• CABG patients with multi-vessel coronary artery disease (including urgent and off pump CABG patients)

#### Exclusion criteria

• Single graft

• Redo CABG

• Evolving myocardial infarction

• Concomitant valve surgery

### Randomisation and enrolment process

All patients requiring CABG should be considered for entry into the study. Centres should keep a screening log and record if the patient is entered into the study and if not the reason why not. Patients who meet the eligibility criteria and give written informed consent should be randomised.

Randomisation will be performed by telephone contact with the trial co-ordinating centre (the Clinical Trials and Evaluation Unit (CTEU) based at the Royal Brompton Hospital in London). The randomisation service will be available 09:00–17:00 (UK time). Centres will be asked for a few simple details about the patient including initials, date of birth and eligibility criteria. The caller will be given the procedure allocation (SIMA or BIMA) and a fax will be sent to the centre confirming this.

Eligible patients will be randomised in equal proportions between the two surgical strategies SIMA or BIMA. The allocated procedure will be performed by a trial nominated surgeon who has been approved by the Trial Steering Committee as being sufficiently experienced (ie. performed >50 BIMA procedures).

Randomisation will be stratified by centre with specific tables using block allocation to provide treatment distribution in equal proportions. The block size will itself be subject to random variation.

To reduce the possibility of outcome measure events occurring after randomisation and before revascularisation, surgery should be performed within 6 weeks of randomisation.

### Surgical procedure

It is left to the individual centres to decide the optimal anaesthetic technique and method of myocardial protection for each patient. As there is a consistent mortality of around 2.5% for CABG across most UK centres this suggests that minor differences in anaesthetic technique and methods of myocardial protection do not have a major influence on perioperative mortality. Surgical details will be recorded in the case report form (CRF). The only requirement is that the surgeon follows the randomisation allocation given for the patient (ie. SIMA or BIMA).

Surgery can be performed with cardiopulmonary bypass or as an off-pump procedure according to the experience of the surgeon.

The following surgical procedures should be applied depending on the allocation to SIMA or BIMA:-

**SIMA **– standard operation using SIMA to LAD plus supplemental vein or radial artery graft

**BIMA **– both IMA placed to left sided coronary arteries with supplemental vein or radial artery. The IMA grafts can be used as composite grafts to each other, as long as one remains *in situ*.

Possible combinations include

• LIMA to OM and RIMA to LAD

• LIMA to LAD and RIMA to OM (via transverse sinus)

• LIMA to LAD and RIMA as composite graft to OM

Some typical examples of SIMA and BIMA grafts are shown in Figure 1.

Please note that anastomosis of an IMA graft to the right coronary artery is an exclusion criteria (because of evidence of inferior long-term patency).

### Outcome measures

#### Primary

• Death from any cause (cardiovascular and non-cardiovascular mortality).

*This outcome will be recorded using the flagging system of the Office of National Statistics (using name, address, date of birth and NHS number)*.

#### Secondary

Secondary clinical outcome measures will be assessed in a blinded fashion by the Clinical Events Review Committee. These outcomes will be measured in hospital, at routine 6-week clinical follow-up and from telephone questionnaires. Outcomes are as follows:-

##### a. Cause-specific death

• Death will be classified into cardiac, other vascular and non-cardiovascular, where possible, using autopsy reports and death certificates. Death will also be classified by ICD code.

• ***Death due to cardiac causes: ****Cardiac causes of death such as congestive heart failure, arrhythmia or myocardial infarction*.

• ***Other vascular causes of death: ****Vascular causes of death such as pulmonary embolus, dissection, cerebrovascular accident or bleeding event*.

• ***Non-cardiovascular causes of death: ****This includes any other cause of death*.

##### b. 30 day mortality

• Death from any cause by 30 days post surgery

• Cause-specific death by 30 days post surgery

##### c. 90 day mortality

• Death from any cause by 90 days post surgery

• Cause-specific death by 90 days post surgery

##### d. Need for re-intervention

ie percutaneous coronary intervention or redo surgery

##### e. Other clinical events

• Myocardial infarction, major bleeding, cerebrovascular accident and other serious adverse events will be captured.

##### f. Quality of Life and cost effectiveness evaluation

• These outcomes will be measured from questionnaires (Rose -shortened form, EuroQol-5D, SF36 and Health Services Resource use). These outcomes will be assessed blind to the knowledge of which arm the trial the patient is in.

### Follow-up

A summary of the follow up is shown in Table 1.

**Table 1:** Follow-up schedule

	**Pre-op**	**Intra-op**	**Pre-discharge**	**6 wk clinic visit (± 1 m)**	**Annual telephone and postal follow up for 10 years (± 1 m)**
***Baseline characteristics***	***√ ***				
***Clinical history***	***√ ***				
***Physical exam***	***√ ***			***√ ***	
***Medications***	***√ ***		***√ ***	***√ ***	***√ ***
***LV function assessment***	***√ ***				
***Quality of Life assessment ***	***√ ***				***√ ***
***Health Service Resource use***		***√ ***	***√ ***	***√ ***	***√ ***
***Operation details***		***√ ***			
***Clinical Outcomes***			***√ ***	***√ ***	***√ ***

Annual Quality of Life questionnaires (Table 2) will be sent to study participants by post, no clinic visits are required apart from the routine clinical 6-week post operative visit. Participants will be sent stamped addressed envelopes to improve the return rates of postal questionnaires. Study co-ordinators will telephone participants to alert them to the questionnaires arrival and to ask them about medications, adverse events and health services resource use.

**Table 2:** Quality of Life questionnaires

**Questionnaire**	**Information collected**	**YEAR**										
		
		Pre-op	1	2	3	4	5	6	7	8	9	10
Rose (shortened form)	Simple, evaluates angina	+	+	+	+	+	+	+	+	+	+	+
EuroQuol-5D	Short and simple, for economic evaluation	+	+	+	+	+	+	+	+	+	+	+
SF-36	Detailed, examines eight separate dimensions	+					+					+

### Health service research issues

Information will be collected in each centre on resources used during the hospital stay, time in operating theatre, total blood and coagulant product use, time in cardiac recovery unit, days on ward; treatment of complications (eg return to theatre), drugs prescribed at hospital discharge, use of cardiac rehabilitation.

Information on subsequent in-patient episodes (including interventions and duration) on outpatient visits and diagnostic procedures, and on general practitioner and practice nurse consultations will be obtained from the patient during the annual telephone call. This call will also ask about specified medications (eg aspirin, statins, ace-inhibitors, beta-blockers, calcium channel antagonists). Participating centres will also record and report subsequent rehospitalisations and revascularisations of patients in the trial.

### Trial size

#### Number of patients

To achieve an absolute 5% reduction in 10-year mortality (ie from 25% to 20%), with 90% power at 5% alpha requires 2928 patients. The mortality data is taken from a summary of previous studies published in reference 12.

The aim is to enrol at least 3000 patients (1500 in each arm) over a 2 to 3 year recruitment period in 20 centres in the UK, Australia, Poland and Brazil (Table 3). As the intervention is the operation, compliance is likely to be 100% except in the unusual situation where the planned operation is not possible for technical reasons.

**Table 3:** ART Principal Investigators and centres

***UK centres***
Mr A Forsyth, Royal Sussex County Hospital, Brighton
Mr A Ritchie, Papworth Hospital, Cambridge
Mr P O'Keefe, University Hospital Of Wales, Cardiff
Mr V Zamvar, Edinburgh Royal Infirmary, Edinburgh
Mr A Cale, Castle Hill Hospital, Hull
Mr T Spyt, Glenfield Hospital, Leicester
Mr W Dihmis, Cardiothoracic Centre, Liverpool
Mr J Gaer, Harefield Hospital, London
Mr J Desai, King's College Hospital, London
Professor J Pepper, Royal Brompton Hospital, London
Mr V Chandrasekaran, St. George's Hospital, London
Mr R Hasan, Manchester Royal Infirmary, Manchester
Mr S Clark, Freeman Hospital, Newcastle
Professor D Taggart, John Radcliffe Hospital, Oxford
Mr N Briffa, Northern General Hospital, Sheffield
***Australian centre***
Professor B Buxton, Austin & Repatriation Medical Centre, Victoria
***Polish centres***
Professor A Bochenek, 1^st ^Dept of Cardiac Surgery, Katowice
Professor S Wos, 2^nd ^Dept of Cardiac Surgery, Katowice
Dr R Pawlaczyk, Medical University of Gdansk, Gdansk
***Brazilian centre***
Dr F Moraes, Heart Institute of Pernambuco, Recife

#### Loss to follow-up

• For the primary outcome (ie death of trial patients) there should be minimal loss as patient death will be automatically flagged via the Office of National Statistics

• For secondary outcome measures around 5% of patients may be lost to follow-up due to non-compliance with questionnaires or movement to other areas. Study co-ordinators at each centre will maintain telephone contact with participants to record Health Service Resource Use, to record adverse events, to alert them of the questionnaires' arrival, to ensure addresses are current and to follow-up any non-responders.

### Statistical issues

#### Type of analyses

The trial data will be analysed on an intention-to-treat basis with patients included in the groups assigned at randomisation, irrespective of future management and events. Outcome measure event tracking will begin at randomisation and continue until death or the end of follow-up. Analysis of time to event data will use log rank tests and possibly other methods suitable for survival data, in particular to take account of known prognostic variables.

#### Frequency of analyses

A limited number of interim analyses will be performed by the trial statistician as specified by the Data Monitoring Committee (DMC). The accumulating results will not be available to the trialists or other principal investigators. The final, definitive analysis of the trial data will be conducted 120 months after the date of commencement of the trial.

#### Analyses of cost-effectiveness and quality of life data

Overall analysis will be performed from the perspective of the health care system; basic information will also be collected on employment status during follow-up from the annual Health Service Resource use questionnaire. Unit costs obtained from centres and from national sources will be used to obtain a cost per patient. Missing data will be handled via multiple imputations in statistical analyses.

In line with the primary outcome of the trial, the main outcome measure in the cost-effectiveness analysis will be life years gained. These will be estimated within-trial and over the patients' lifetime, by taking the sum of life years obtained in each arm of the trial within the follow-up period and modelling subsequent life expectancy, using different assumptions about any within-trial treatment effects continuing. The cost per quality adjusted life year (QALY) will be calculated in the same way, using results from the EQ-5D annual questionnaires.

The cost-effectiveness analysis will be reported in terms of the incremental cost per life year and the incremental cost per QALY gained. All resource use, cost, outcome and cost-effectiveness information will be reported as the mean per patient in each arm of the trial and the mean difference, with appropriate measures of variance. Cost-effectiveness in sub-groups will be estimated by applying any overall relative risk reduction and cost reduction to different baseline absolute risk groups. Cost-effectiveness acceptability curves and net benefit statistics will also be reported.

#### Sub-group analyses

i. Diabetic vs nondiabetic

ii. Age > 70 years vs age <70 years

iii. On-pump vs off-pump

iv. Radial vs vein grafts

v. Number of grafts

vi. Left ventricular failure

### Trial organisation

#### Trial Steering Committee (TSC)

The main role of the TSC is to monitor and supervise the progress of the trial. The TSC membership is listed in Table 4. Independent members are Professor Vermes (patient lay member), Professor Sleight (Chairman and cardiologist), Dr Stables (cardiologist), Ms Farrell (trials advisor). The TSC will meet prior to the start of the trial and then annually or as required thereafter.

**Table 4:** Trial Steering Committee membership

**Name**	**Trial Role**	**Title**	**Affiliation**
Professor G Vermes	Patient Lay Member	Emeritus Professor of Hebrew Studies	Oxford
Professor D Altman	Statistician	Professor of Statistics in Medicine,	Oxford
Professor J Dark	Lead Surgeon	Professor of Cardiac Surgery	Newcastle
Ms B Farrell	Trials Advisor	Co-Director, Resource Centre for Randomised Trials	Oxford
Dr M Flather	Co-Principal Investigator	Director, CTEU, Royal Brompton Hospital	London
Professor A Gray	Health Economist	Professor of Health Economics	Oxford
Professor J Pepper	Lead Surgeon	Professor of Cardiac Surgery	London
Professor P Sleight	CHAIRMAN	Emeritus Professor Cardiology	Oxford
Professor K Channon	Cardiologist	Professor of Cardiovascular Surgery	Oxford
Dr R Stables	Independent Cardiologist	Consultant Cardiologist	Liverpool
Professor D Taggart	Chief Investigator	Consultant Cardiac Surgeon	Oxford

#### Data Monitoring Committee (DMC)

The main role of the DMC is to consider the data from any interim analyses and specifically to assess any safety issues (such as unexpected serious adverse events) that occur and report back to the TSC. The DMC membership is listed in Table 5. All members of the DMC are independent of the trial. The DMC will meet at the start of the trial and then annually or as required thereafter. The DMC will be expected to develop, in agreement with the investigators, a charter outlining their responsibilities and operational details.

**Table 5:** Data Monitoring Committee membership

**Name**	**Trial Role**	**Title**	**Affiliation**
Professor S Yusuf	CHAIRMAN	Consultant Cardiologist	Hamilton
Professor S Pocock	Statistician	Professor of Medical Statistics	London
Professor D Julian	Cardiology advisor	Emeritus Professor of Cardiology	London
Professor T Treasure	Surgical advisor	Professor of Cardiothoracic Surgery	London

#### Clinical Event Review Committee

The Clinical Event Review Committee will review adverse events during the study and adjudicate them to ensure the events meet the definitions given. Each event will be independently adjudicated by two committee members. A third committee member will be called to adjudicate an event if agreement is not reached by two members. These assessments will be blind to the knowledge of which arm of the trial the patients are in.

#### Study co-ordination

The study will be co-ordinated and managed by the Clinical Trials and Evaluation Unit (CTEU) a dedicated clinical trials department within the Royal Brompton Hospital. In addition to providing overall project co-ordination, the CTEU will assist in preparing the final protocol, the investigators' manuals, design the Case Report Forms (CRF), provide the randomisation service and design and instigate the data management system. The CTEU will ensure that the trial runs according to the pre-agreed timetable, recruitment targets are met, CRFs are completed accurately and that all aspects of the study are performed to the highest quality. The CTEU will also assist in the training of investigators and co-ordinators at the start-up of the study and for performing monitoring procedures throughout.

The trial management team from CTEU will meet the Chief Investigator weekly by conference call.

### Centres

#### Study sites

The list of participating centres is given in Table 3. Each centre will be paid a pro-rata sum of 1.0 FTE for years 1–2, 0.5 FTE for year 3 and 0.2 FTE for years 4–10, to help support a study co-ordinator.

Study site co-ordinators will be responsible for screening patients (and recording the data on a screening log), enrolling patients into the trial, providing a contact point for patients, liasing with CTEU, completing CRFs, ensuring annual questionnaires are sent and processed, recording adverse events, ensuring forms are sent to CTEU and that all edit queries are resolved.

### Data collection

Each centre will be provided with a Protocol, Manual of Operations, questionnaires and patient CRFs. Data will be recorded onto two part NCR CRFs and the top copy sent to the CTEU at the times specified. Specific adverse event forms for death, myocardial infarction, major bleed, cerebrovascular accident, revascularisation and other serious adverse events (ie. other events that require or prolong hospitalisation) are provided. Centres are required to complete these adverse event forms and fax to the CTEU within 72 hours of their knowledge of the event.

### Investigators' responsibilities

Surgeons must have completed a minimum of 50 BIMA operations before commencing on the study. Investigators must ensure that Local Ethics Committee approval has been obtained as well as Agreements signed off by their Institution prior to the start of the study.

Investigators are required to ensure compliance to the protocol, CRFs and Manual of Operations. Investigators are required to allow access to study documentation or source data on request for monitoring visits and audits performed by the CTEU or any regulatory authorities.

### Training

#### Pre-study training visit

Before the study commences each centre will receive a training visit by CTEU. These visits will ensure that personnel at each site (including principal investigators, co-investigators and the study site co-ordinator) fully understand the protocol, CRF and the practical procedures for the study.

#### Monitoring visits

At regular intervals during the study CTEU will perform monitoring visits to each centre. The purpose of these visits is to ensure compliance to the protocol and that ethical and regulatory guidelines are met. Source data verification and checking of essential documents will be performed. Monitoring visits also provide an opportunity for further training if required (eg new staff). Central review of study data will also be performed throughout the study.

#### Close-out visit

At the end of the study each centre will receive a site visit from CTEU to resolve any outstanding edit queries or adverse events and to verify the correct storage of study documentation.

### Ethics

This study will conform to the MRC Guidelines for Good Clinical Practice in Clinical Trials (1998) [30] and the Declaration of Helsinki guidelines (2004) [31]. The study protocol will be submitted to the appropriate Ethical Review Committee in each country participating in the study and approval will be obtained before the study commences.

### Informed consent

"Informed consent" requires individual discussion with the patient about the nature of the procedures to be conducted in a language that is easy to comprehend. The patient should fully understand that he/she might be allocated to either the SIMA or the BIMA group. The patient should also understand that his/her refusal to participate in the study will not affect the quality of subsequent medical care. It is recommended that, if possible, the patient has at least 24 hours to think about participation and discussing with family or friends before signing the consent form.

Before any trial-related procedures may be performed, informed consent must be obtained from the patient by the investigator by means of a signed declaration. The investigator must sign in the CRF to confirm that informed consent was obtained and store the original of the signed declaration of consent in the patient's notes. A copy should be given to the patient and a copy filed in the patient's CRF.

### Publication policy

The results from the trial will be submitted for publication in a major journal irrespective of the outcome. The Trial Steering Committee will be responsible for approval of all manuscripts arising from the study prior to submission for publication. Sub-studies of centre-specific data may only be carried out with the knowledge and approval of the Trial Steering Committee.

Authorship of presentations and reports related to the study will be in the name of the collaborative group. The final follow-up study results paper will name local co-ordinators as well as those involved in central co-ordination and trial management. Co-ordinators who provided data will be named. Certificates of collaboration will be provided to those who have made a substantial contribution but whose name is not on the final report.

Papers on other aspects of the study will be published with those who made substantive contributions being named as authors. These papers will make appropriate acknowledgement of the contribution of the collaborativegroup. At the end of the study, patients will be able to request a copy of the results of the study from the investigator at that site.

### Proposed timetable for ART study

Jun 2004 Grant awarded by MRC & BHF - Recruitment starts

Jun 2007 Recruitment completed

2009 First patient completes 5 years

2012 Last patient completes 5 years  - Analysis and publication of 5 year data

2014 First patient completes 10 years

2017 Last patient completes 10 years

2017/8 Publication of 10 year data

## Discussion

The ART study is one of the first randomised trials to evaluate the effects on survival and other clinical outcomes of SIMA grafting versus BIMA grafting, and will help to establish the best approach for patients requiring CABG surgery.

## Abbreviations

CABG: coronary artery bypass graft

IMA: internal mammary artery

SIMA: single internal mammary artery

BIMA: bilateral internal mammary artery

OPCAB: off-pump coronary artery bypass

LIMA: left internal mammary artery

RIMA: right internal mammary artery

LAD: left anterior descending artery

OM: obtuse marginal branch of the circumflex coronary artery

CRF: case report form

## Competing interests

The author(s) declare that they have no competing interests.

## Authors' contributions

DPT: conceived of the study and participated in the design and co-ordination and helped to draft the manuscript

BL: participated in the design and co-ordination and helped to draft the manuscript

AG: participated in the design and the health economic analysis and helped to draft the manuscript

DA: participated in the design and statistical analysis helped to draft the manuscript

MF: participated in the design and helped to draft the manuscript

KC: participated in the design and helped to draft the manuscript

All authors read and approved the final manuscript.

## Study support

ART is funded jointly by a grant from the British Heart Foundation (SP/03/001) and a grant from the Medical Research Council (G0200390). The BHF and MRC peer-reviewed the grant application and have approved the manuscript submission.

## Source of funding for authors

Each author is supported directly by their host institution.
